# Enamel Hypoplasia and Dental Fluorosis in Children With Special Healthcare Needs: An Epidemiological Study

**DOI:** 10.7759/cureus.36440

**Published:** 2023-03-20

**Authors:** Soham M Brahmbhatt, Anurag Rawat, Ankita Sharma, Ayeesha Urooge, Sidhant Pathak, Debajyoti Bardhan

**Affiliations:** 1 Public Health Dentistry, AMC Dental College and Hospital, Gujarat University, Ahmedabad, IND; 2 International Cardiology, Himalayan Institute of Medical Sciences, Dehradun, IND; 3 Pedodontics and Preventive Dentistry, Private Practitioner, Amritsar, IND; 4 Oral Medicine and Radiology, MAK Multi-Speciality Dental Care, Bangalore, IND; 5 Dentistry, Adesh Medical College & Hospital, Shahbad, IND; 6 Oral Medicine and Radiology, Hi-Tech Medical College & Hospital, Bhubaneswar, IND

**Keywords:** hypoplasia, children, needs, health care, dental fluorosis

## Abstract

Background

Children with cerebral palsy, intellectual difficulties, or hearing deficiencies may have enamel hypoplasia. Moreover, as the child begins to walk, the fluorosis progresses to bone malformations in the lower limbs, and it fully manifests as the child grows up.

Methodology

An epidemiological study was conducted to assess the prevalence of dental caries, oral hygiene status, enamel opacities, and oral hygiene habits among children 4-15 years of age from various schools for special healthcare needs including government schools and non-government organizations.

Results

The study sample comprised 1,047 children with special healthcare needs in the age group of 4-15 years, with 608 males and 439 females. It was seen that 29.79% of vegetarian children were affected with caries, while only 16.14% of children with a mixed diet were affected with caries.

Conclusions

There is no significant association between enamel hypoplasia and the physical and mental disabilities of children with special healthcare needs.

## Introduction

Children who have or are at increased risk for a long-term physical, developmental, behavioral, or close-to-home condition and require medical services related to administrations of a kind beyond that expected by children in general are a significant population in need of medical care administrations, as defined by the Maternal and Youngster Health Branch (MCHB) [1]. Oral pathologies are more pronounced in children with disabilities for several reasons such as medical. Caries may be caused by economic and social factors, as well as by self-mutilating habits (such as frequent tooth grinding) and the cariogenic action of drugs with a high sugar content [2]. Parents or guardians are usually responsible for these children performing routine activities including oral care [3].

Locations with naturally high fluoride concentrations in drinking water are prone to endemic dental fluorosis. Clinically, the disease manifests as dull, opaque white patches or striations in the enamel. Too much fluoride consumed over a long period of time during tooth growth causes hypomineralization [4]. Water content in fluoride is responsible for causing both dental and skeletal fluorosis [5]. However, the real status of this element in the body cannot be completely reflected by the fluorine content in food and water. There is evidence that this condition is less common among vegetarians compared to non-vegetarians [6,7].

Primary enamel abnormalities may be inherited but many children also develop enamel hypoplasias as a consequence of illness throughout infancy, most often from infections, metabolic imbalances, preterm delivery, or malnutrition [8]. It is quantitative in nature and manifests as a deficient thickness of enamel [9]. Children with cerebral palsy, intellectual difficulties, or hearing problems are disproportionately affected by enamel hypoplasia. This finding suggests that systemic changes that affect brain development may potentially affect tooth germ growth [10]. Hydrofluorosis affects a large proportion of the population in India [2]. It affects approximately 18 of the 33 states in the country. About 62 million individuals, including about 6 million children, in India, have fluorosis owing to drinking fluoridated water [5]. In infants and toddlers (aged 0-1 year), the initial symptom is frequent discomfort while walking or running, followed by pain when the child is weaned from breast milk and given a normal diet and water [6]. As the kid learns to walk, the condition presents itself more severely, causing abnormalities in the lower extremities [11]. The prevalence of endemic dental fluorosis is higher in regions where fluoride levels in drinking water are higher. White, opaque spots, or striations on the enamel are a clinical hallmark of this illness. The enamel is chipped in some instances and tinted a yellowish-brown in numerous others. Commonly, lesions appear on both sides of the body in the same pattern [4].

In general, the teeth and gums of persons with disabilities are just as robust and healthy as those of typically developing individuals. However, their dental health is negatively affected by several variables, including nutrition, eating patterns, medication, physical restrictions, lack of cleaning practices, and the attitudes of parents and health professionals [12]. To reduce the likelihood of biofilm-dependent, avoidable illnesses, it is crucial to study the prevalence of enamel hypoplasia and fluorosis in children with special healthcare requirements, as well as the risk factors associated with them. The oral health of normal children has been the subject of several studies. Comparing the oral health of children receiving various types of special care has not been extensively studied. By comparing the dental health of children in various groups that need special care, we can better understand their requirements and provide recommendations for improving their oral health. This research aimed to examine dental caries and oral hygiene practices among 7-17-year-old children in Rajasthan’s orphanages and foster care facilities.

## Materials and methods

Children aged 4-15 attending government and non-government organization (NGO) schools for children with special healthcare needs in the Jodhpur district were surveyed as part of an epidemiological study to determine the prevalence of dental caries, oral hygiene status, deft, enamel opacities, and oral hygiene habits. Before beginning the survey, we obtained permission and informed consent from the legal guardians of the students and the school principals in charge of the special education programs in the Jodhpur area’s Department of Education.

Age, gender, diet, and the kind of disability were noted for each participant before the dental examination. All children included in the study had been diagnosed with developmental disabilities. All children were from families who gave permission to participate in the research. All children were from special schools. Subjects were excluded if they lacked the cognitive abilities necessary to participate during an oral assessment. Each individual was classified as either blind, deaf, or dumb; intellectually disabled; orthopedically disabled; or a member of more than one of these groups. Oral hygiene index-simplified (OHI-S) where selective six index teeth were included and scored for debris and calculus which were then added to get the index score, Dental Caries (WHO criteria), fluorosis, and dentition status were used to evaluate patients’ oral health status in accordance with the WHO standards [13].

After examining each kid, we documented our results in a Performa we made using WHO criteria and gave them to the classroom instructors at the end of the day. The information was converted from a pre-coded survey Performa file to an Excel spreadsheet. To facilitate the examination of the collected data, a master file was developed. We tallied the descriptive data such as mean, SD, and percentages over all the different groups. The chi-square and analysis of variance tests were used to evaluate the data.

## Results

The study sample comprised 1,047 children with special healthcare needs in the age group of 4-15 years, with 608 males and 439 females. It was observed that 0.57% of the participants were blind and 4.68% were orthodontically impaired, requiring help from others in maintaining oral hygiene (Tables 1-3).

**Table 1:** Sex-wise distribution of the study population in different special schools.

Serial number	Males	Females	Total
School 1	105	63	168
School 2.	86	64	150
School 3	39	31	70
School 5	47	27	74
School 6.	26	14	40
School 7	38	29	67
School 8	184	165	349
School 9	73	56	129
Grand total	608	439	1,047

**Table 2:** Distribution of the study population among handicapped groups according to sex.

Handicapped groups	Males	Females	Total
Blind	119 (11.37%)	94 (8.97%)	213 (20.34%)
Deaf and dumb	185 (17.67%)	145 (13.85%)	330 (31.52%)
Orthopedically impaired	167 (15.95%)	103 (9.84%)	270 (25.79%)
Intellectual disability	68 (6.50%)	49 (4.68%)	117 (11.18%)
Cerebral palsy	8 (0.76%)	5 (0.47%)	13 (1.24%)
Multiple disabilities	61 (5.82%)	43 (4.10%)	104 (9.93%)
Total	608 (58.09%)	439 (41.91%)	1,047 (100%)

**Table 3:** Distribution of the study population according to the mode of cleaning teeth.

Handicapped groups	Themselves	Other’s help	Under supervision	Total
Blind	189 (18.05%)	6 (0.57%)	18 (1.72%)	213 (20.34%)
Deaf and dumb	330 (31.52%)	0 (0.00%)	0 (0.00%)	330 (31.52%)
Orthopedically impaired	221 (21.11%)	49 (4.68%)	0 (0.00%)	270 (25.79%)
Intellectual disability	21 (2.00%)	64 (6.11%)	32 (3.06%)	117 (11.18%)
Cerebral palsy	3 (0.29%)	1 (0.09%)	9 (0.85%)	13 (1.24%)
Multiple disabilities	74 (7.07%)	6 (0.57%)	24 (2.29%)	104 (9.93%)
Total	838 (80.03%)	126 (12.03%)	83 (7.94%)	1,047 (100%)

Furthermore, 29.79% of vegetarian children were affected by caries, while only 16.14% of children with a mixed diet were affected by caries. The oral hygiene status of handicapped children with a vegetarian diet (30%) was poor compared to children with a mixed diet (41%). Moreover, oral hygiene status among females was better compared to males (Tables 4, 5).

**Table 4:** Caries prevalence of the study population according to diet.

Sex	Vegetarian		Mixed	
	Subject	Caries affected	Subject	Caries affected
Males (n = 608)	405 (66.6%)	168 (27.63%)	203 (33.38%)	88 (14.4%)
Females (n = 439)	310 (70.6%)	144 (32.8%)	129 (29.38%)	81 (18.45%)
Total (n = 1,047)	715 (68.29)	312 (29.79%)	332 (31.7%)	169 (16.14%)

**Table 5:** Oral hygiene status of the study population according to diet among handicapped groups.

Handicapped groups	Vegetarian				Mixed			
	Good	Fair	Poor	Total	Good	Fair	Poor	Total
Blind	39	96	50	185	07	09	12	28
Deaf and dumb	47	92	67	206	35	48	41	124
Orthopedically impaired	45	67	52	164	15	24	57	106
Intellectual disability	23	32	22	77	09	17	14	40
Cerebral palsy	4	01	01	06	01	04	02	07
Multiple disabilities	24	24	29	77	06	08	13	27
Total	182	312	221	715	83	110	139	332

Dental fluorosis

Out of 156 blind students, 21 (2%) had mild fluorosis, while 21 students had questionable fluorosis out of 287 deaf and dumb children. Six (0.57%) orthopedically impaired students out of 234 suffered from severe fluorosis. Moreover, 1.15% of students with intellectual disabilities had a questionable status of fluorosis. Out of nine children suffering from cerebral palsy, two had mild fluorosis. On the other hand, 12 out of 82 children with multiple disabilities were affected with very mild fluorosis (Table 6).

**Table 6:** Dental fluorosis.

Handicap group	Normal	Questionable	Very mild	Mild	Moderate	Severe	Excluded	Not recorded
Blind	156	8	19	21	1	8	0	0
Deaf and dumb	287	21	9	12	0	1	0	0
Orthopedically impaired	234	18	7	2	3	6	0	0
Intellectual disability	96	12	8	0	0	1	0	0
Cerebral palsy	9	1	1	2	0	0	0	0
Multiple disabilities	82	9	12	0	0	1	0	0
Total	864	69	56	37	4	17	0	0

Enamel hypoplasia

Out of 195 blind students, eight had demarcated opacity while seven had hypoplasia. Eleven students out of 297 deaf and dumb students and 14 orthopedically impaired students were affected with hypoplasia. The teeth of nine children with intellectual disabilities out of 98 were affected by hypoplasia. Moreover, 17 out of 80 children with multiple disabilities were affected by hypoplasia (Table 7).

**Table 7:** Enamel hypoplasia.

Handicapped groups	Normal	Demarcated opacity	Diffuse opacity	Hypoplasia	Other defects	Demarcated and diffuse opacity	Demarcated opacity and hypoplasia	Diffuse opacity	All three conditions	Not recorded
Blind	195	8	1	7	0	1	0	0	1	0
Deaf and dumb	297	12	5	11	0	1	0	1	3	0
Orthopedically impaired	235	19	1	14	0	0	0	0	1	0
Intellectual disability	98	4	2	9	0	0	3	1	0	0
Cerebral palsy	12	0	0	0	0	1	0	0	0	0
Multiple disabilities	80	7	0	17	0	0	0	0	0	0
Total	917	50	9	58	0	3	3	2	5	0

## Discussion

Due to the growing tooth germ’s susceptibility and inability to recover from a variety of systemic disruptions, the tooth enamel often functions as a reservoir of information on systemic insults acquired during development. There is an increased risk of enamel defects in primary teeth for children with cerebral palsy, mental retardation, and sensory-neural hearing loss, among other neurologic impairments [14,15]. Both the primary (baby) teeth and the permanent (grown-in) teeth may be affected by these factors, which can start at any time before or after birth. Typically, permanent teeth are impacted, especially the teeth that are still developing at the time of the disruption [16].

A study by Clarkson and O’Mullane [17] claimed that the age gap between the eight-year-olds and the 15-year-olds provided a sample size large enough to detect trends in the occurrence of abnormalities on both early and late erupting teeth. Jindal also found that, among children with disabilities, the frequency of enamel developmental abnormalities was significant. This demonstrates the link between many systemic disorders and tooth formation [16]. Developmental defects of enamel were shown to be prevalent in both primary and permanent teeth in a study conducted in Himachal [18]. Goodman, who conducted his research in 1991, also reported a link between enamel hypoplasia and poor nutrition [19].

Caries susceptibility, wear, sensitivity, and cosmetic issues are all factors dentists need to watch out for when treating patients with enamel hypoplasia. Children with special needs, such as cerebral palsy, mental retardation, or hearing loss, are more likely to have primary teeth affected by enamel hypoplasia [20]. However, no such specific association was observed in this study between developmental anomaly and enamel hypoplasia. Similar results were reported by Basha et al. [21].

Because drinking water is the most common way for people to get their daily dose of fluoride, it is commonly believed that doing so can reduce the prevalence of tooth decay, especially in young children. However, consuming too much fluoride in drinking water can lead to harmful effects, such as dental fluorosis [22]. Chemicals such as fluorides have the potential to disrupt mineralization and calcium homeostasis, both of which are necessary for proper enamel development [15]. In this study, no significant results were observed between disabled children and dental fluorosis.

In the present study, 29.79% of the children with a vegetarian diet were affected by caries while only 16.14% of the children with a mixed diet were affected by caries. The oral hygiene status of handicapped children with a vegetarian diet (30%) was poor compared to children with a mixed diet. Similar results were reported by Reddy and Sharma [23] and Saravanakumar et al. [24] in their study of children with special healthcare needs [25]. Vegetarianism was linked to increased odds of tooth erosion, as this study pointed out. The high frequency of caries and periodontal disease is linked to factors including the use of sweet drugs and the difficulties of biofilm management by children with special healthcare requirements [26]. Hence, studying the developmental defects of enamel and the variables that contribute to its prevalence in people with cerebral palsy is crucial for lowering the risk of avoidable illnesses reliant on biofilms.

However, there were a few limitations of this research. Dentifrice ingestion while brushing may have been underestimated due to recall bias associated with the questionnaire, which collected information on the duration of pregnancy, the timing of delivery, and the child’s health history. Neither typically developing nor medically fragile children were included in the analysis of oral hygiene practices.

## Conclusions

Those who are deaf, blind, or otherwise visually impaired often feel they are not given accurate information regarding their diagnosis, treatment choices, and prognosis. However, the results of the current research show that enamel hypoplasia is not linked to developmental delays or other health issues among children who need special healthcare services. Clinicians need to tailor their approaches to each patient because of the wide range of circumstances that might influence the impairments of these patients.

## References

[1] van Dyck PC, Kogan MD, McPherson MG, Weissman GR, Newacheck PW (2004). Prevalence and characteristics of children with special health care needs. Arch Pediatr Adolesc Med.

[2] Purohit BM, Singh A (2012). Oral health status of 12-year-old children with disabilities and controls in southern India. WHO South East Asia J Public Health.

[3] Singh A, Kumar A, Berwal V, Kaur M (2014). Comparative study of oral hygiene status in blind and deaf children of Rajasthan. J Adv Med Dent Sci.

[4] Shyama M, Al-Mutawa SA, Honkala S (2001). Prevalence of dental fluorosis in disabled children and young adults in Kuwait. Med Principles Pract.

[5] Cao J, Liu JW, Tang LL (2005). Dental and early-stage skeletal fluorosis in children induced by fluoride in brick-tea. Fluoride.

[6] Galchenko AV, Sherstneva AA (2022). Fluorine status among vegans, vegetarians and religious fasters. p. 358-63.

[7] Awadia AK, Haugejorden O, Bjorvatn K, Birkeland JM (1999). Vegetarianism and dental fluorosis among children in a high fluoride area of northern Tanzania. Int J Paediatr Dent.

[8] Pascoe L, Seow WK (1994). Enamel hypoplasia and dental caries in Australian aboriginal children: prevalence and correlation between the two diseases. Pediatr Dent.

[9] Robles MJ, Ruiz M, Bravo-Perez M, González E, Peñalver MA (2013). Prevalence of enamel defects in primary and permanent teeth in a group of schoolchildren from Granada (Spain). Med Oral Patol Oral Cir Bucal.

[10] Modrić VE, Verzak Ž, Karlović Z (2016). [Developmental defects of enamel in children with intellectual disability]. Acta Stomatol Croat.

[11] Khandare AL, Harikumar R, Sivakumar B (2005). Severe bone deformities in young children from vitamin D deficiency and fluorosis in Bihar-India. Calcif Tissue Int.

[12] Sihag TP, Ahmed A, Ojha A, Singh S, Patil SR, Alam MK (2022). Evaluation of oral health status of children with special health care needs in Jodhpur district of India. Int Med J.

[13] (2022). World Health Organization. Oral health surveys—basic methods. https://apps.who.int/iris/handle/10665/41905.

[14] Nunn JH, Murray JJ (1987). The dental health of handicapped children in Newcastle and Northumberland. Br Dent J.

[15] Bhat M, Nelson KB (1989). Developmental enamel defects in primary teeth in children with cerebral palsy, mental retardation, or hearing defects: a review. Adv Dent Res.

[16] Jindal C, Palaskar S, Kler S (2011). The prevalence of the developmental defects of enamel in a group of 8-15 years old Indian children with developmental disturbances. J Clin Diagn Res.

[17] Clarkson J, O'Mullane D (1989). A modified DDE Index for use in epidemiological studies of enamel defects. J Dent Res.

[18] Chauhan D, Chauhan T (2013). Prevalence of developmental defects of enamel in mixed and permanent dentition of 9 and 12 year old children of Himachal Pradesh, India: a cross sectional study. Int J Health Allied Sci.

[19] Goodman A, Jerome R (1991). Dental enamel hypoplasias as indicators of nutritional status. Adv Dent Anthrop.

[20] Slayton RL, Warren JJ, Kanellis MJ, Levy SM, Islam M (2001). Prevalence of enamel hypoplasia and isolated opacities in the primary dentition. Pediatr Dent.

[21] Basha S, Mohamed RN, Swamy HS (2014). Prevalence and associated factors to developmental defects of enamel in primary and permanent dentition. Oral Health Dent Manag.

[22] Mandinic Z, Curcic M, Antonijevic B, Carevic M, Mandic J, Djukic-Cosic D, Lekic CP (2010). Fluoride in drinking water and dental fluorosis. Sci Total Environ.

[23] Reddy K, Sharma A (2011). Prevalence of oral health status in visually impaired children. J Indian Soc Pedod Prev Dent.

[24] Saravanakumar M, Vasanthakumari A, Bharathan R (2013). Oral Health status of special health care needs children attending a day care centre in Chennai. Int J Stud Res.

[25] Smits KP, Listl S, Jevdjevic M (2020). Vegetarian diet and its possible influence on dental health: a systematic literature review. Community Dent Oral Epidemiol.

[26] Nogueira BR, Silva AM, de Castelo Branco Araújo T, Ferreira MC, Mendes RF, Prado Júnior RR (2021). Exploring the association of predisposing factors of cerebral palsy and developmental defects of enamel: a case-control study. Eur Arch Paediatr Dent.

